# Reliability of ultrasound assessment of the rectus femoris muscle thickness: intra-rater, inter-rater, and inter-day analysis accounting for age and sex

**DOI:** 10.1186/s12891-024-08033-5

**Published:** 2024-11-15

**Authors:** Martin Bjørn Stausholm, Katielle Rodrigues da Silva, Pedro Augusto Inácio, Alberto Souza de Sá Filho, Pedro Sardinha Leonardo Lopes-Martins, Jan Magnus Bjordal, Patrícia Sardinha Leonardo, Rodrigo Alvaro B. Lopes-Martins

**Affiliations:** 1 Department of Global Public Health and Primary Care, University of Bergen, Bergen, Hordaland Norway; 2 Laboratory of Biophotonics and Experimental Therapeutics, Universidade Evangélica de Goiás, UniEvangelica. Av. Universitária Km 3,5, Anápolis, Goiás Brazil; 3 Integrated Laboratory of Neurosciences and Physical Conditioning, Universidade Evangélica de Goiás, UniEvangelica. Av. Universitária Km 3,5, Anápolis, Goiás Brazil; 4 Laboratório de Tecnologias em Saúde, Universidade Evangélica de Goiás, UniEvangelica. Av. Universitária Km 3,5, Anápolis, Goiás Brazil; 5 Post-Graduate Program in Bioengineering, Universidade Brasil, Av. Carolina Fonseca 236, Itaquera, São Paulo, Brazil

**Keywords:** Muscle thickness, Ultrasonography, Observer variation, Reliability

## Abstract

**Background:**

The rectus femoris muscle plays an important role in maintaining lower limb stability and biomechanical control during basic physical activities. Both reduced quadriceps strength and decreased muscle thickness of the rectus femoris, as measured by ultrasound, are associated with an increased risk of falls in older adults. We estimated the relative and absolute intra-rater, inter-rater, and inter-day reliability of the procedure.

**Methods:**

A female biologist and a male physical educator, both holding a master’s degree in human movement and rehabilitation, captured ultrasound images of the right rectus femoris muscle in 106 healthy participants (58 females and 48 males), aged 18 to 73 years. Each rater captured three images per participant during each visit, with two visits 7 to 10 days apart. A third person subsequently measured the muscle thickness. We calculated the Intraclass Correlation Coefficients (ICC) using a two-way random model and determined the 95% minimal detectable difference (MDD).

**Results:**

The mean muscle thickness was 2.12 cm. The reliability based on single measurements was as follows: the intra-rater ICC for raters A and B was 0.998 at both visits (MDDs: 0.074–0.082 cm). The inter-rater ICC was 0.976 at visit 1 and 0.977 at visit 2 (MDDs: 0.269–0.270 cm). The inter-day ICC was 0.973 for rater A and 0.972 for rater B (MDDs: 0.286–0.291 cm). Sensitivity analyses accounting for age, sex, and the use of mean measurements produced similar results. The results were homoscedastic.

**Conclusions:**

The rectus femoris muscle thickness was measured with good reliability using ultrasonography across all the settings.

**Supplementary Information:**

The online version contains supplementary material available at 10.1186/s12891-024-08033-5.

## Introduction

In humans, muscle mass remains relatively stable during early life, but after the age of 30, a natural process of muscle mass reduction begins at a rate of 0.5 to 1.0% per year [1]. With the aging process, the impaired balance between protein synthesis and proteolysis in skeletal muscle results in a progressive decline in skeletal muscle mass, strength, and function, which is referred to as sarcopenia [2]. On average, peak muscle strength decreases by 20 to 40% between the ages of 30 and 80 [3].

Strength training is considered the most effective strategy for the prevention and treatment of sarcopenia [4]. Randomized controlled trials have shown positive effects of strength training on muscle mass, muscle strength, and physical performance [5, 6].

The rectus femoris muscle is a vital component of the quadriceps group, serving as a powerful knee extensor and contributing to hip flexion [7]. Therefore, the rectus femoris muscle plays an important role in maintaining lower limb stability and biomechanical control during activities such as walking, running, jumping, and stair climbing [7]. Both reduced quadriceps strength and decreased muscle thickness (MT) of the rectus femoris, as measured by ultrasound, are associated with an increased risk of falls in older adults [8, 9]. The simultaneous assessment of muscle strength and size contributes to a more comprehensive understanding of muscle physiology and potential for improvement [8].

Assessment of reliability is a necessary first step in the validation process of clinical tests [10]. The reliability of ultrasound measurements is influenced by the standardization of anatomical location, pressure force applied during the assessment, and rater experience [11]. The reliability of the measurement has been evaluated in a series of studies, but their sample sizes were relatively small. The inter-rater reliability has been investigated in 12 healthy individuals by Takahashi et al. [12], in 15 healthy individuals and 17 patients with chronic obstructive pulmonary disease by Hammond et al. [11], in 29 critically ill patients by Pardo et al. [13], and in 99 hospitalized patients by Pinto-Ramos et al. [14]. The inter-rater intra-class correlation coefficient (ICC) point estimates ranged between 0.70 and 0.98 (relative reliability where 1 is perfect). Despite test-retest reliability being crucial for confirming the consistency of measurements over time, the evidence on the subject is very sparse. The inter-day ICC has been estimated to be 0.88 by Lima et al., based on 15 healthy individuals [15] and 0.97 by Thomaes et al., based on 25 patients with coronary artery disease [16].

Therefore, we conducted a new study to assess the relative and absolute intra-rater, inter-rater, and inter-day reliability of the procedure with a larger sample.

## Methods

The study was approved by the Human Research Ethics Committee of the Universidade Evangélica de Goiás (UniEvangélica), according to CEP opinion no. 6.210.982, with Certificate of Submission for Ethical Appraisal (CAAE) no. 69796523.7.0000.5076. All research procedures were conducted in accordance with the principles outlined in the Declaration of Helsinki. Those who agreed to participate signed a Free and Informed Consent Form (TCLE), in accordance with resolution 196/96 of the National Health Council (CNS).

### Participants

The study included 106 participants of both sexes, aged between 18 and 73 years, with preserved cognitive ability and no other significant illness identified through a clinical history, blood pressure, and pulse measurements, physical examination, and complementary tests. They were recruited from the city of Anápolis – Goiás between August and December 2023.

### Ultrasonography procedure

The ultrasonography was conducted using a Mindray M6 B-mode ultrasound device equipped with a linear transducer (model L14-6Ns) operating at a frequency of 10 megahertz (MHz). The dynamic range (DR.70) and gain (G.50) were kept constant for all measurements. The depth was adjusted between 3.7 and 4.6 cm to ensure clear visualization of the rectus femoris muscle and its superficial and deep aponeuroses.

Two raters performed the ultrasonography: a 36-year-old female biologist with a master’s degree in human movement and rehabilitation (rater A) and a 28-year-old male physical educator with a master’s degree in human movement and rehabilitation (rater B). Prior to the study, the raters completed an ultrasonography course and spent 3 months practicing the specific procedures at the university clinics.

The participants were positioned supine on a stretcher with their legs fully extended and muscles relaxed for 10 min. Anatomical landmarks, including the greater trochanter and lateral epicondyle of the femur of the right thigh, were identified for each participant. The MT of the rectus femoris was analyzed at a point located 40% of the femur’s length proximal to these landmarks [7]. Meticulous skin markings were made at each visit, using a non-permanent pen, to ensure standardized distances between anatomical points and consistent transducer positioning. During the procedure, a generous amount of ultrasound gel, soluble in contact water, was applied. The gel was directly applied to the transducer to minimize image distortion and facilitate the acoustic coupling necessary for accurate measurements. Measurements were exclusively performed on the participants’ right legs. Each rater captured a total of three images per participant for analysis at each visit, with 2-3-minute intervals that did not include probe-to-skin contact. All participants underwent two visits scheduled 7 to 10 days apart. All sessions took place during the same period of the day in an air-conditioned room with only one rater present at the time.

### Image processing

**Fig. 1:**
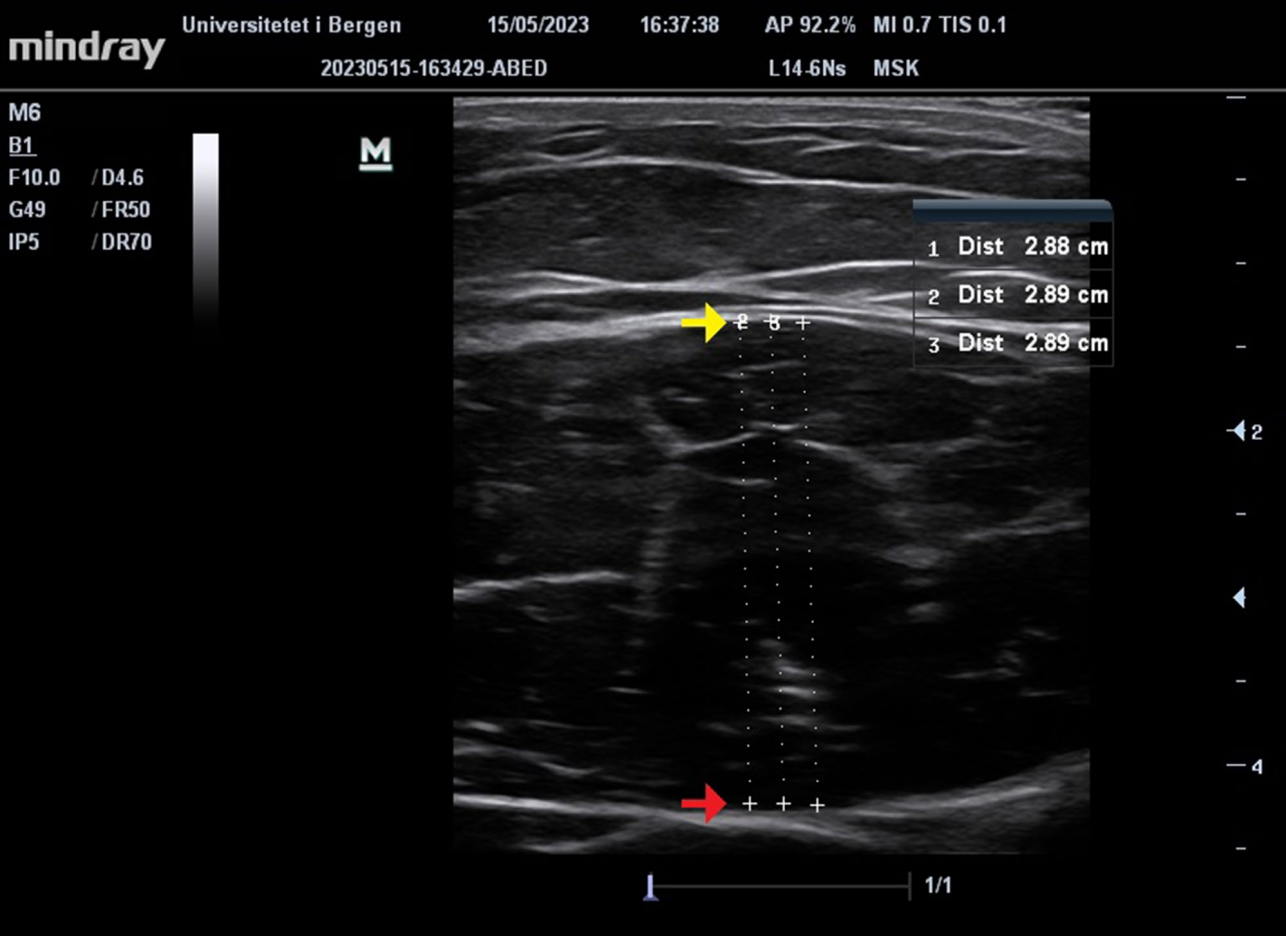
Example of an ultrasound image being processed. The yellow arrorw marks the the superficial aponeurosis, and the red arrow marks the inferior aponeurosis

An ultrasound technician, not otherwise involved in the study, measured the MT using the software integrated into the ultrasound device. The MT was defined as the distance between the superficial aponeurosis and the deepest aponeurosis. Measurements were taken at three different locations, and the average distance was calculated for analysis (Fig. 1).

### Statistics

Descriptive statistics were applied using IBM SPSS Statistics 29 and Microsoft Excel 2016.

Intra-rater reliability was estimated using the first and second measurements (image number 1 and 2). Inter-rater and inter-day reliability were estimated using the first measurements (image number 1), as well as the means of three measurements (all 3 images).

Relative reliability was calculated utilizing the ICC two-way random model with average measures and absolute agreement. The ICC values were interpreted as proposed by JC Nunnally and IH Bernstein [17]: ICC values of ≥ 0.900 represent good reliability.

Absolute reliability was calculated using within-subject standard deviation (S_w_); the difference between a measurement and the true value can be expected to be less than 1.96*S_w_ for 95% of observations. The Minimal Detectable Difference (MDD) in centimeter MT that must be exceeded to be 95% confident that a real change has occurred between measurements was estimated using the formula S_w_*1.96*√2 [18]. The distribution of data was inspected using Bland-Altman plots showing the means and differences of paired measurements, along with the 95% limits of agreement [19].

## Results

The characteristics of the participants are described in Table 1.

**Table 1:** Sex, age, and rectus femoris muscle thickness of the participants

	Females (*n* = 58)		Males (*n* = 48)		Both sexes (*n* = 106)	
	Age (years)	MT (cm)	Age (years)	MT (cm)	Age (years)	MT (cm)
Mean	29.91	1.86	36.00	2.43	32.67	2.12
Min-max	18–73	0.96–2.68	18–67	1.54–3.25	18–73	0.96–3.25
SD	14.00	0.32	14.08	0.38	14.27	0.45

The numbers for MT are based on measurement 1 and 2 by both raters. MT = Muscle thickness; SD = Standard deviation

### Overall analysis

**Fig. 2:**
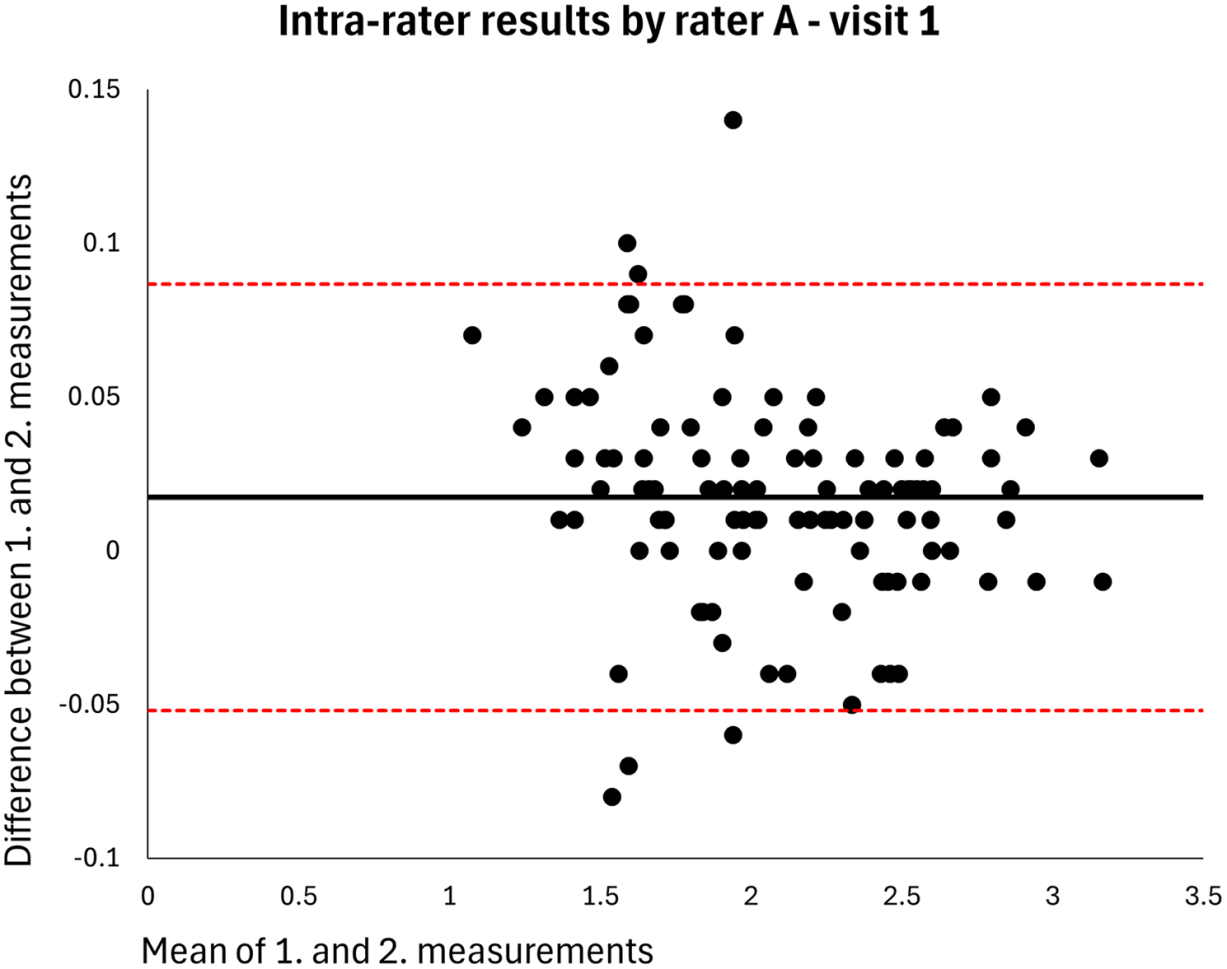
Level of agreement between rater A’s 1. and 2. measurements at visit 1. The values are cm. The thick horizontal solid line represents the mean difference and the dotted horizontal lines represents the 95% limits of agreement

**Fig. 3:**
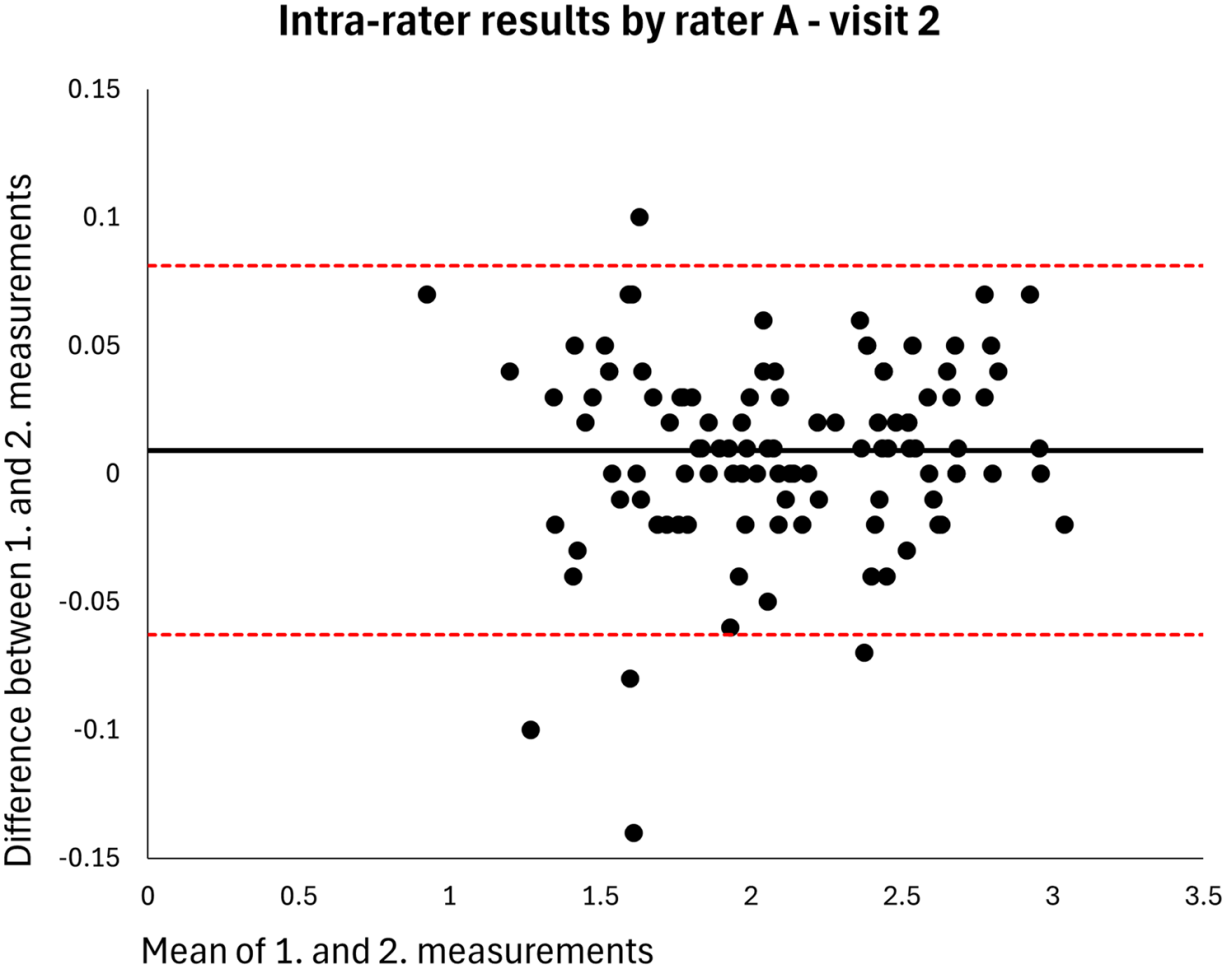
Level of agreement between rater A’s 1. and 2. measurements at visit 2. The values are cm. The thick horizontal solid line represents the mean difference and the dotted horizontal lines represents the 95% limits of agreement

**Fig. 4:**
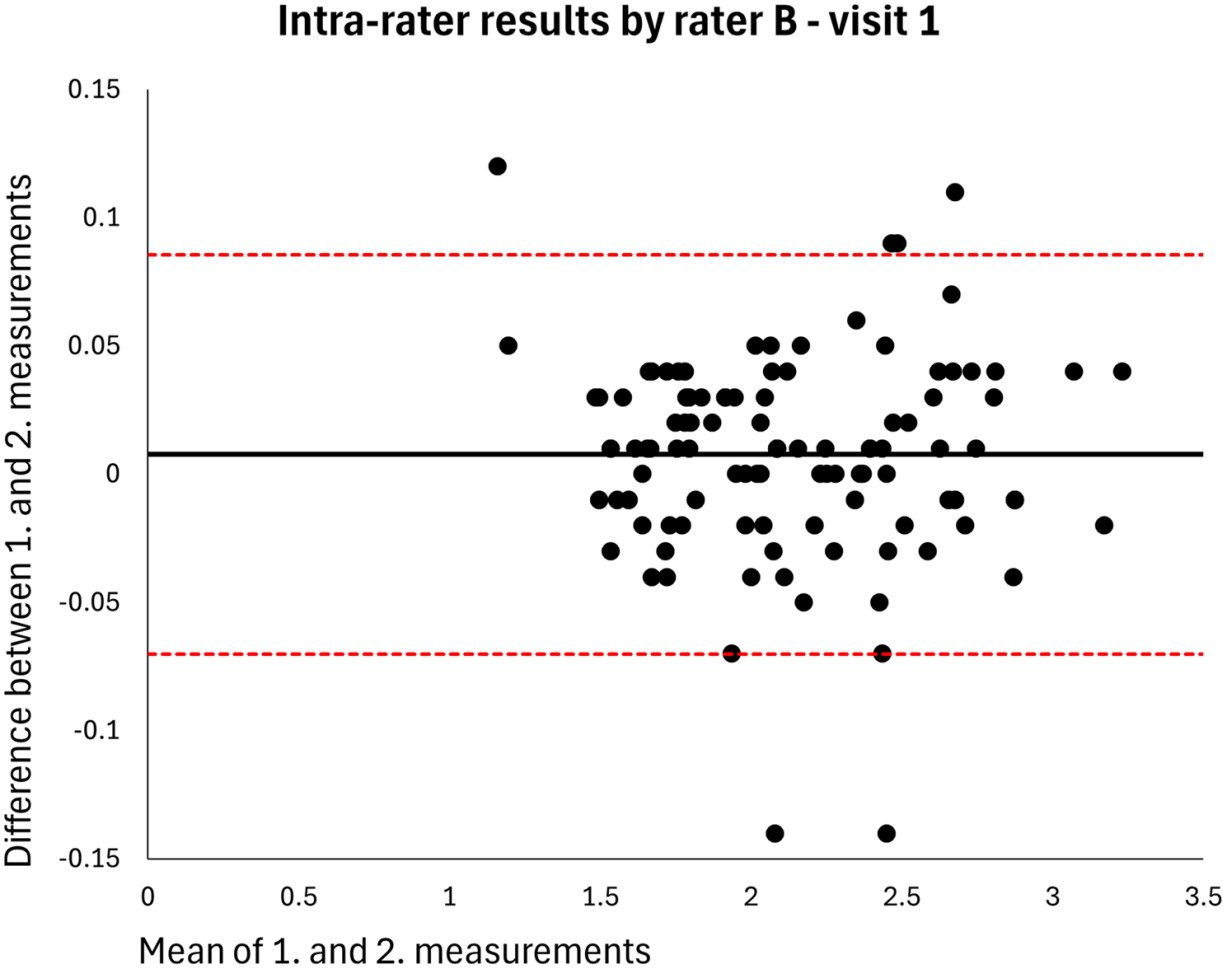
Level of agreement between rater B’s 1. and 2. measurements at visit 1. The values are cm. The thick horizontal solid line represents the mean difference and the dotted horizontal lines represents the 95% limits of agreement

**Fig. 5:**
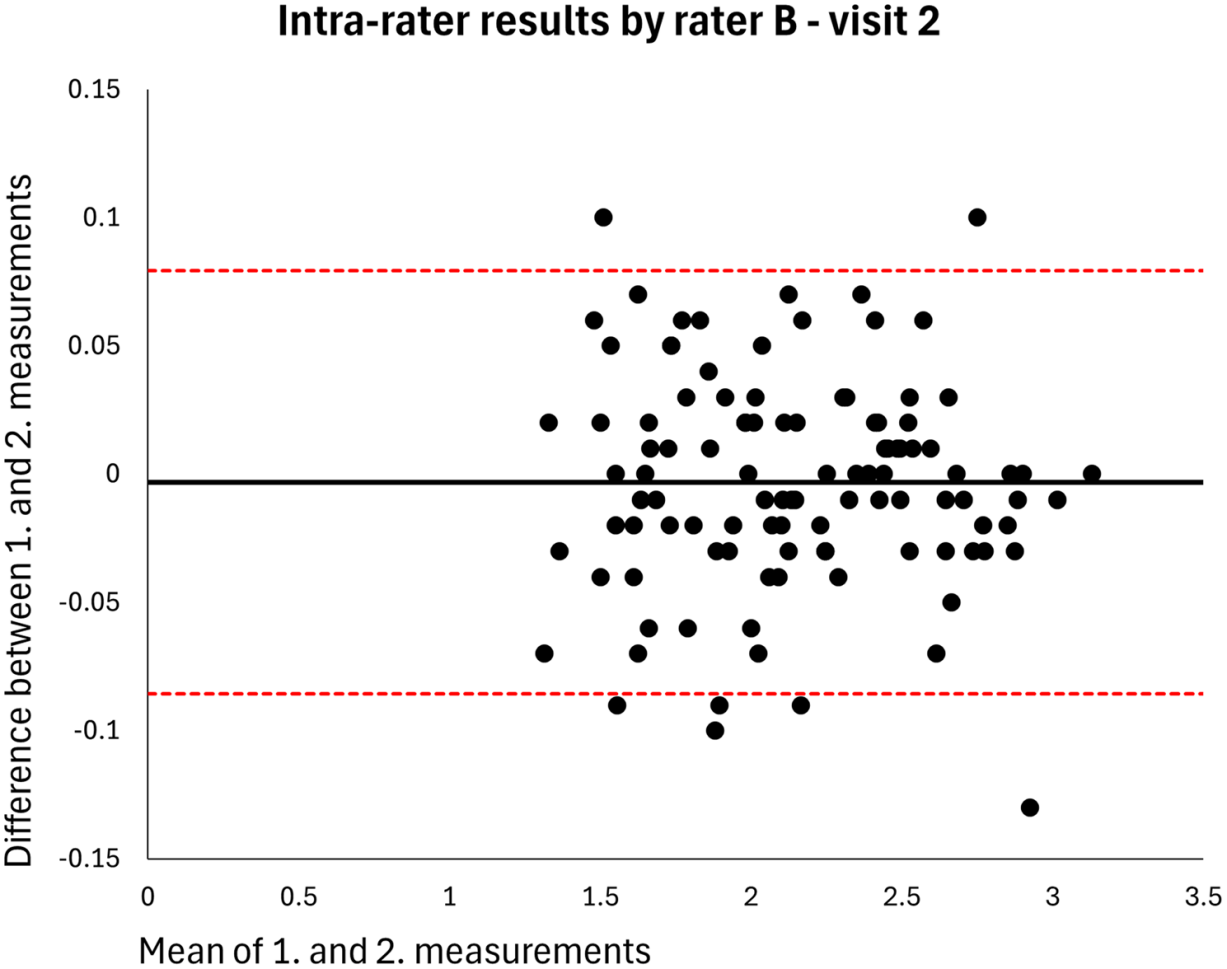
Level of agreement between rater B’s 1. and 2. measurements at visit 2. The values are cm. The thick horizontal solid line represents the mean difference and the dotted horizontal lines represents the 95% limits of agreement

**Fig. 6:**
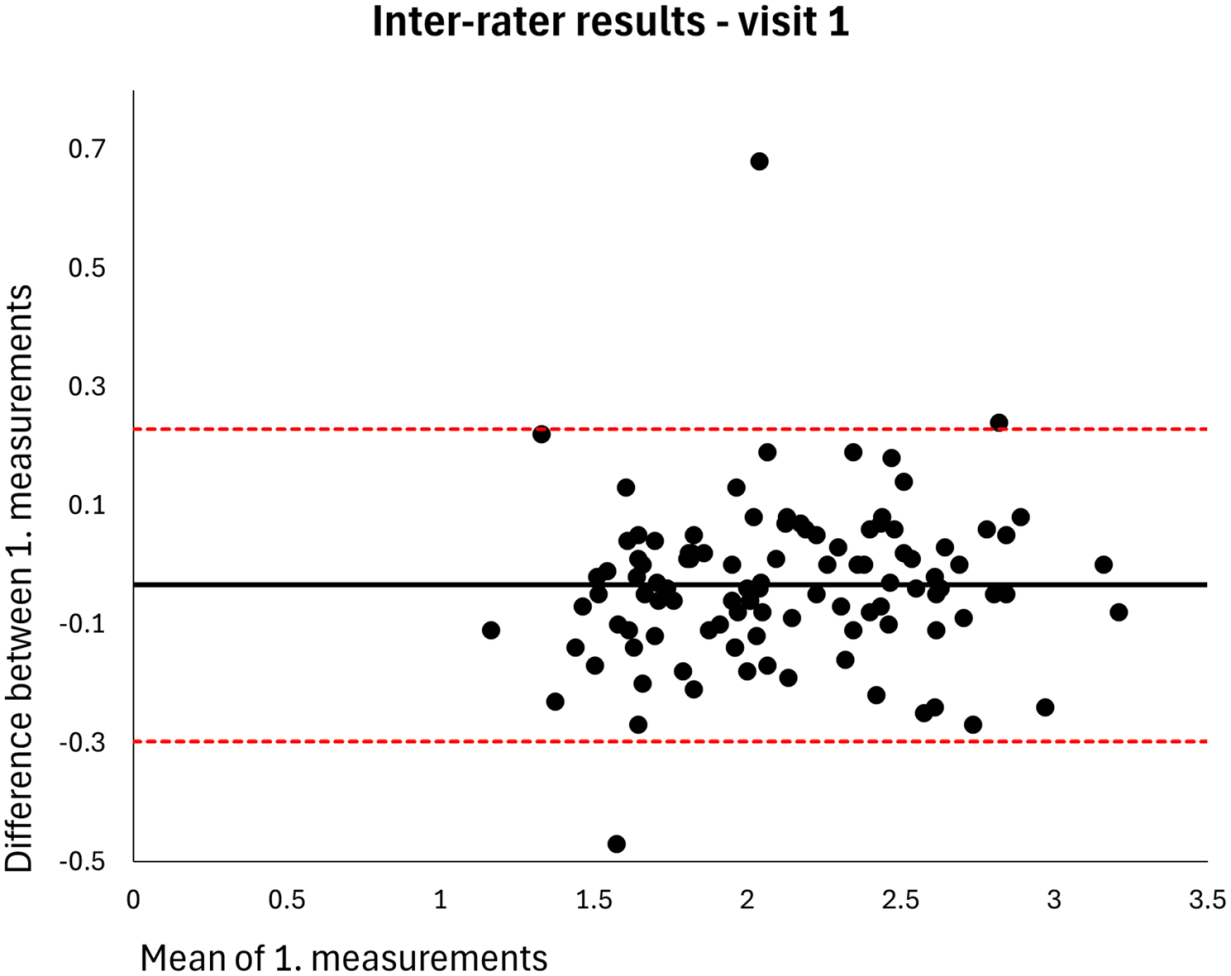
Level of agreement between rater A’s and B’s 1. measurements at visit 1. The values are cm. The thick horizontal solid line represents the mean difference and the dotted horizontal lines represents the 95% limits of agreement

**Fig. 7:**
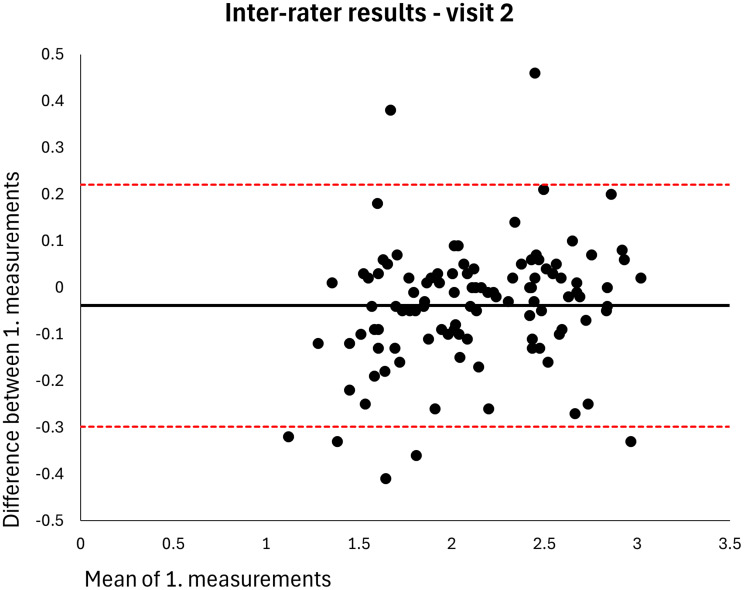
Level of agreement between rater A’s and B’s 1. measurements at visit 2. The values are cm. The thick horizontal solid line represents the mean difference and the dotted horizontal lines represents the 95% limits of agreement

**Fig. 8:**
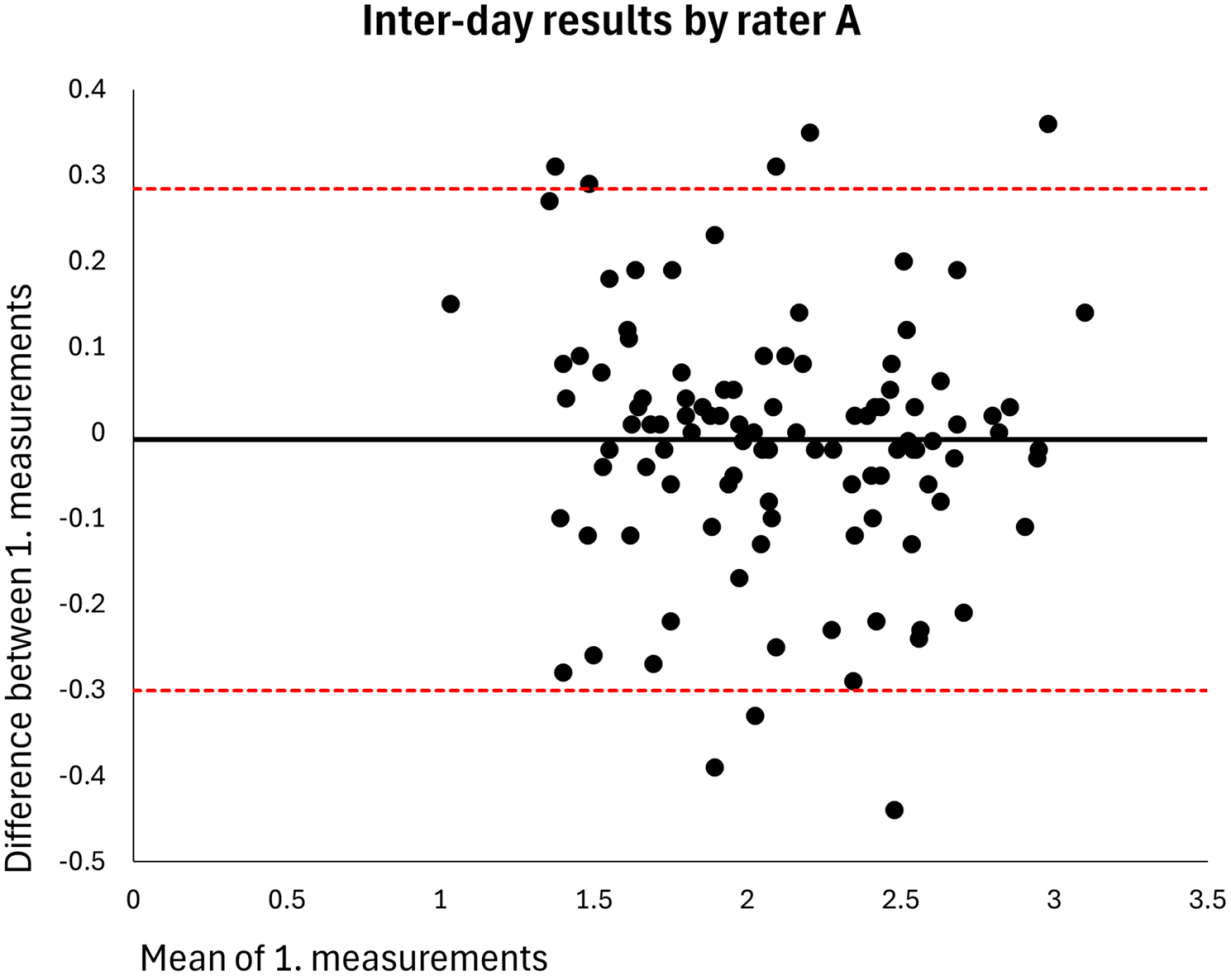
Level of agreement between rater A’s 1. measurements at visit 1 versus at visit 2. The values are cm. The thick horizontal solid line represents the mean difference and the dotted horizontal lines represents the 95% limits of agreement

**Fig. 9:**
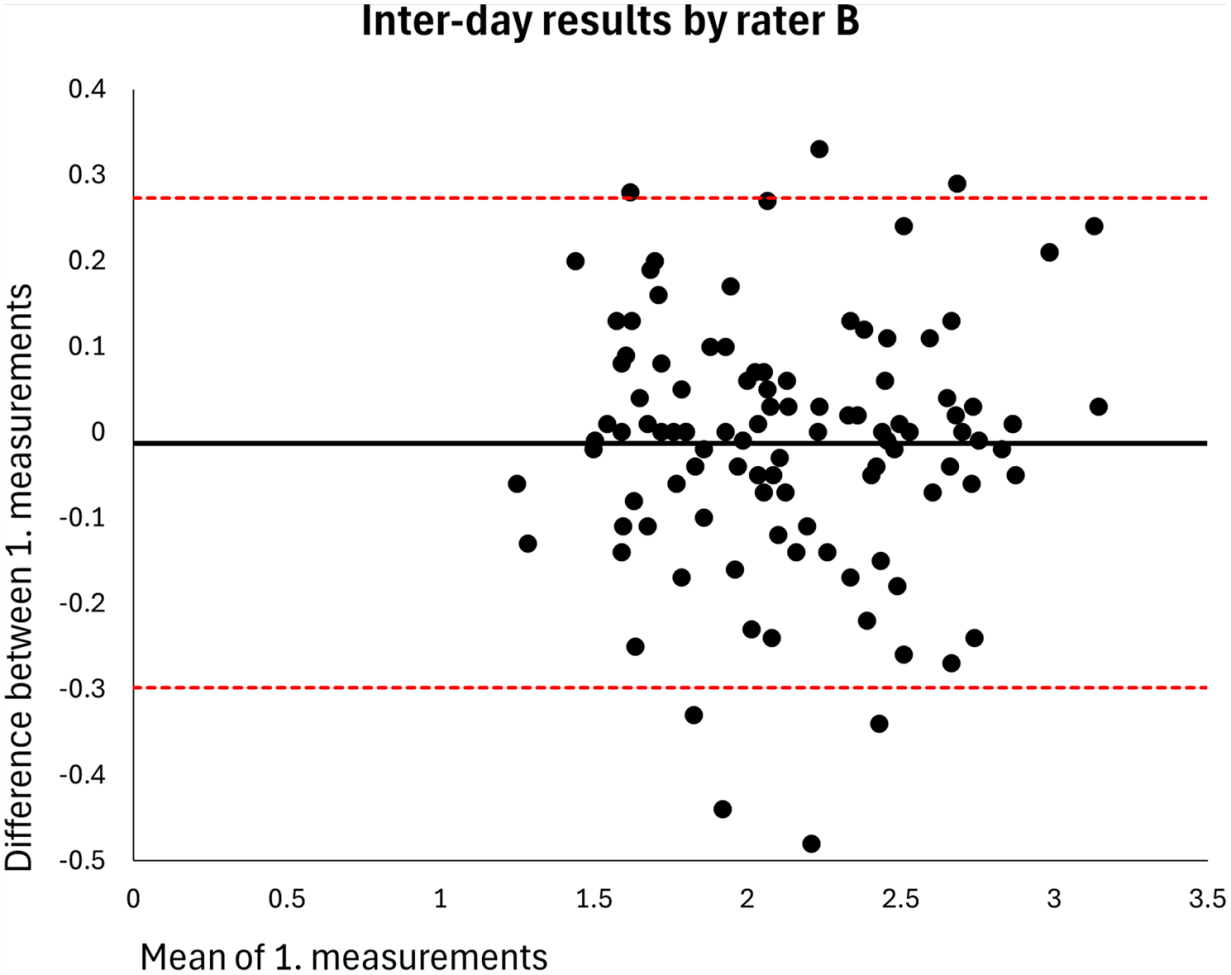
Level of agreement between rater B’s 1. measurements at visit 1 versus at visit 2. The values are cm. The thick horizontal solid line represents the mean difference and the dotted horizontal lines represents the 95% limits of agreement

The mean MT of the entire sample was 2.120 cm (*n* = 106). The intra-rater ICC for raters A and B was 0.998 at both visits (MDDs = 0.074–0.082 cm) (Table 2). Based on single measurements, the inter-rater ICC was 0.976 at visit 1 and 0.977 at visit 2 (MDDs = 0.269–0.270 cm) and the inter-day ICC was 0.973 for rater A and 0.972 for rater B (MDDs = 0.286–0.291 cm) (Table 2). The results were homoscedastic according to the Bland-Altman plots (Figs. 2, 3, 4, 5, 6, 7, 8 and 9). The results based on means of three measurements were not meaningfully different (Table 2).

**Table 2:** Agreement between single measurements and between means of measurements in all participants

Rater	ICC (95% CI)	Mean (cm)	95% CI of true value (cm)	MDD (cm)	MDD vs. mean (%)
Intra-rater reliability – agreement between 1. and 2. measurements – visit 1					
A	0.998 (0.996–0.999)	2.089	± 0.054	0.077	3.7
B	0.998 (0.997–0.999)	2.128	± 0.056	0.079	3.7
Intra-rater reliability – agreement between 1. and 2. measurements – visit 2					
A	0.998 (0.998–0.999)	2.100	± 0.052	0.074	3.5
B	0.998 (0.997–0.998)	2.146	± 0.058	0.082	3.8
Inter-rater reliability – agreement between 1. measurements – visit 1					
AB	0.976 (0.963–0.984)	2.114	± 0.191	0.270	12.8
Inter-rater reliability – agreement between means of 3 measurements – visit 1					
AB	0.978 (0.967–0.985)	2.107	± 0.181	0.256	12.1
Inter-rater reliability – agreement between 1. measurements – visit 2					
AB	0.977 (0.964–0.985)	2.125	± 0.190	0.269	12.7
Inter-rater reliability – agreement between means of 3 measurements – visit 2					
AB	0.977 (0.962–0.985)	2.120	± 0.189	0.267	12.6
Inter-day reliability – agreement between 1. measurements					
A	0.973 (0.961–0.982)	2.101	± 0.206	0.291	13.9
B	0.972 (0.958–0.981)	2.138	± 0.202	0.286	13.4
Inter-day reliability – agreement between means of 3 measurements					
A	0.976 (0.964–0.983)	2.094	± 0.196	0.277	13.2
B	0.973 (0.961–0.982)	2.133	± 0.196	0.278	13.0

CI = Confidence Interval; ICC = Intraclass Correlation Coefficient; MDD = Minimal Detectable Difference

### Analysis of participants under the age of 50 years

The mean MT of the participants under the age of 50 years was 2.143 cm (*n* = 91). The intra-rater ICC for raters A and B was 0.998 at both visits (MDDs = 0.073–0.081 cm) (Supplemental Table 1). Based on single measurements, the inter-rater ICC was 0.975 at visit 1 and 0.976 at visit 2 (MDDs = 0.257–0.259 cm) and the inter-day ICC was 0.967 for rater A and 0.970 for rater B (MDDs = 0.288–0.304 cm) (Supplemental Table 1).

### Analysis of participants over the age of 50 years

The mean MT of the males was 1.977 cm (*n* = 15). The intra-rater ICC for rater A was 0.999 at both visits (MDDs = 0.079–0.080 cm) and the intra-rater ICC for rater B was 0.999 at visit 1 and 0.998 at visit 2 (MDDs = 0.081–0.089 cm) (Supplemental Table 2). Based on single measurements, the inter-rater ICC was 0.977 at visit 1 and 0.976 at visit 2 (MDDs = 0.331–0.336 cm) and the inter-day ICC was 0.992 for both raters (MDDs = 0.195 cm) (Supplemental Table 2).

### Analysis of female participants

The mean MT of the females was 1.861 cm (*n* = 58). The intra-rater ICC for rater A was 0.995 at visit 1 and 0.997 at visit 2 (MDDs = 0.075–0.089 cm) and the intra-rater ICC for rater B was 0.996 at visit 1 and 0.995 at visit 2 (MDDs = 0.078–0.086 cm) (Supplemental Table 3). Based on single measurements, the inter-rater ICC was 0.939 at visit 1 and 0.942 at visit 2 (MDDs = 0.297–0.303 cm) and the inter-day ICC was 0.944 for rater A and 0.950 for rater B (MDDs = 0.294–0.309 cm) (Supplemental Table 3).

### Analysis of male participants

The mean MT of the males was 2.432 cm (*n* = 48). The intra-rater ICC for rater A was 0.999 at visit 1 and 0.998 at visit 2 (MDDs = 0.060–0.072 cm) and the intra-rater ICC for rater B was 0.997 at both visits (MDDs = 0.078–0.080 cm) (Supplemental Table 4). Based on single measurements, the inter-rater ICC was 0.977 at visit 1 and 0.976 at visit 2 (MDDs = 0.223–0.233 cm) and the inter-day ICC was 0.967 for rater A and 0.970 for rater B (MDDs = 0.255–0.269 cm) (Supplemental Table 4).

## Discussion

In this study, a 36-year-old female biologist and a 28-year-old male physical educator, both with a master’s degree in human movement and rehabilitation, assessed the rectus femoris MT in healthy persons using ultrasonography to estimate the intra-rater, inter-rater, and inter-day reliability of the procedure. The raters followed a standardized assessment protocol, which they had practiced extensively prior to the study. We estimated the reliability based on both single measurements and means of three consecutive measurements, but the results did not vary meaningfully. The relative reliability was good across all the settings. Overall, the intra-rater MDD corresponded to 3.5–3.7% of the MT, the inter-rater MDD corresponded to 12.7–12.8% of the MT, and the inter-day MT corresponded to 13.4–13.9% of the MT. Interestingly, the percentages were generally slightly higher for female participants and participants over the age of 50 years, which is probably due to their lower MT compared to their counterparts. The only exception was a lower inter-day percentage for the older age group, which ranged from 4.7 to 10.0%. Whether the measurement errors are acceptable depends on the context in which the measurements are being used, including the analytical goals of the user [20, 21]. In a study by Isenmann et al., the rectus femoris MT of middle-aged pre-menopausal women was increased by 0.20 cm (10.1%) over a 10-week conventional strength training program for the upper and lower body [22]. In elderly women, Pinto et al. observed an increase in rectus femoris MT of 0.26 cm (16.1%) over a 6-week conventional leg strength training program [23]. In young adult untrained males, Matta et al. found a 0.29 cm (13.7%) increase in rectus femoris MT over a 14-week conventional upper and lower body strength training regimen [24]. In elderly males, Nogueira et al. observed increases in rectus femoris MT of 0.10 cm (5.3%) and 0.21 cm (11.3%) over a period of 10 weeks with conventional upper and lower body strength training [25]. When interpreting these findings, it is important to consider that exercising over an extended period is likely to yield better results. Also, additional interventions, such as an increase in daily protein intake during a strength training regimen, may further enhance gains in muscle mass [26]. Conversely, a systematic review and meta-analysis by Fazzini et al. showed that critical illness resulted in an average loss of 1.75% of rectus femoris MT per day during the initial week of illness [27]. These results may reflect a catabolic effect owed to immobilization and poor nutrition associated with critical illness [28].

In contrast to our findings, Hammond et al. [11] and Takahashi et al. [12] found that the relative inter-rater reliability of the procedure in healthy individuals was only acceptable. Similarly, Lima et al. [15] reported that the relative inter-day reliability was only acceptable. The discrepancies may be a result of the smaller sample sizes in the three studies (106 versus 12–15 healthy individuals), that is, random error. Variation in rater experience is another plausible explanation [11]. In our study, the ICCs were consistently high across the two visits, indicating that the raters had adequate initial experience.

Our inter-rater ICC values are also higher than those in studies on the topic that included patients [11, 14, 16]. However, it is essential to recognize that ICC values may fluctuate depending on the specific models utilized, and the models used have rarely been reported in studies on the matter. We chose the ICC two-way random model, assuming that both the raters and participants were randomly selected [29].

### Limitations of the study

Only 15 of the participants were over the age of 50 years, which may limit the generalizability of our findings regarding older populations.

## Conclusions

The two raters were able to assess the rectus femoris MT with good intra-rater, inter-rater, and inter-day ICCs using ultrasonography in healthy individuals, consistently across different age and sex groups.

## Electronic supplementary material

Below is the link to the electronic supplementary material.

Supplementary Material 1

## Data Availability

The datasets used and analyzed during the current study are available from the corresponding author on reasonable request.

## Abbreviations

CI: Confidence interval
ICC: Interclass correlation coefficient
MDD: Minimal detectable difference
MHz: Megahertz
MT: Muscle thickness
SD: Standard deviation
Sw: Within-subject standard deviation
