# Supplementary material for: Reliability of ultrasound assessment of the rectus femoris muscle thickness: intra-rater, inter-rater, and inter-day analysis accounting for age and sex
Source: BMC Musculoskelet Disord. 2024 Nov 15;25:916. doi: 10.1186/s12891-024-08033-5 (PMC11566654; doi:10.1186/s12891-024-08033-5)
Supplement: Supplementary file 1 — Supplementary Material 1 [file 12891_2024_8033_MOESM1_ESM.pdf]

Supplementary material for the article:

Reliability of ultrasound assessment of the rectus femoris muscle thickness: intra-rater, inter-rater, and inter-day analysis accounting for age and sex

Martin Bjørn Stausholm, Katielle Rodrigues da Silva, Pedro Augusto Inácio, Alberto Souza de Sá Filho, Pedro Sardinha Leonardo Lopes-Martins, Jan Magnus Bjordal, Patrícia Sardinha Leonardo, Rodrigo Alvaro B. Lopes-Martins

BMC Musculoskeletal Disorders (2024)

Contents

Supplemental Table 1 Agreement between single measurements and between means of measurements in participants under the age of 50 years (n = 91)................................2

Supplemental Table 2 Agreement between single measurements and between means of measurements in participants over the age of 50 years (n = 15) 2

Supplemental Table 3 Agreement between single measurements and between means of measurements in female participants (n = 58) 3

Supplemental Table 4 Agreement between single measurements and between means of measurements in male participants (n = 48) 3

Supplemental Table 1 Agreement between single measurements and between means of measurements in participants under the age of 50 years (n = 91)

Rater	ICC (95% CI)	Mean (cm)	95% CI of true value (cm)	MDD (cm)	MDD vs mean (%)
Intra-rater reliability – agreement between 1. and 2. measurements – visit 1					
A	0.998 (0.996-0.999)	2.111	± 0.054	0.076	3.6
B	0.998 (0.996-0.998)	2.149	± 0.056	0.079	3.7
Intra-rater reliability – agreement between 1. and 2. measurements – visit 2					
A	0.998 (0.997-0.999)	2.131	± 0.052	0.073	3.4
B	0.998 (0.996-0.998)	2.167	± 0.057	0.081	3.7
Inter-rater reliability – agreement between 1. measurements – visit 1					
AB	0.975 (0.962-0.984)	2.135	± 0.183	0.259	12.1
Inter-rater reliability – agreement between means of 3 measurements – visit 1					
AB	0.978 (0.966-0.986)	2.129	± 0.171	0.242	11.4
Inter-rater reliability – agreement between 1. measurements – visit 2					
AB	0.976 (0.964-0.985)	2.151	± 0.182	0.257	11.9
Inter-rater reliability – agreement between means of 3 measurements – visit 2					
AB	0.977 (0.964-0.986)	2.147	± 0.177	0.250	11.6
Inter-day reliability – agreement between 1. measurements					
A	0.967 (0.951-0.979)	2.128	± 0.215	0.304	14.3
B	0.964 (0.946-0.976)	2.159	± 0.216	0.306	14.2
Inter-day reliability – agreement between means of 3 measurements					
A	0.970 (0.955-0.980)	2.120	± 0.204	0.288	13.6
B	0.967 (0.949-0.978)	2.155	± 0.209	0.295	13.7

CI = Confidence Interval; ICC = Intraclass Correlation Coefficient; MDD = Minimal Detectable Difference.

Supplemental Table 2 Agreement between single measurements and between means of measurements in participants over the age of 50 years (n = 15)

Rater	ICC (95% CI)	Mean (cm)	95% CI of true value (cm)	MDD (cm)	MDD vs mean (%)
Intra-rater reliability – agreement between 1. and 2. measurements – visit 1					
A	0.999 (0.995-1.000)	1.953	± 0.056	0.079	4.0
B	0.999 (0.996-1.000)	2.000	± 0.057	0.081	4.1
Intra-rater reliability – agreement between 1. and 2. measurements – visit 2					
A	0.999 (0.996-1.000)	1.920	± 0.056	0.080	4.2
B	0.998 (0.995-0.999)	2.015	± 0.063	0.089	4.4
Inter-rater reliability – agreement between 1. measurements – visit 1					
AB	0.977 (0.932-0.992)	1.987	± 0.234	0.331	16.7
Inter-rater reliability – agreement between means of 3 measurements – visit 1					
AB	0.977 (0.934-0.992)	1.973	± 0.233	0.330	16.7
Inter-rater reliability – agreement between 1. measurements – visit 2					
AB	0.976 (0.910-0.993)	1.967	± 0.237	0.336	17.1
Inter-rater reliability – agreement between means of 3 measurements – visit 2					
AB	0.974 (0.910-0.992)	1.961	± 0.250	0.353	18.0
Inter-day reliability – agreement between 1. measurements					
A	0.992 (0.976-0.998)	1.943	± 0.138	0.195	10.0
B	0.998 (0.994-0.999)	2.011	± 0.067	0.095	4.7
Inter-day reliability – agreement between means of 3 measurements					
A	0.992 (0.978-0.997)	1.935	± 0.138	0.195	10.1
B	0.997 (0.990-0.999)	1.999	± 0.088	0.125	6.3

CI = Confidence Interval; ICC = Intraclass Correlation Coefficient; MDD = Minimal Detectable Difference.

Supplemental Table 3 Agreement between single measurements and between means of measurements in female participants (n = 58)

Rater	ICC (95% CI)	Mean (cm)	95% CI of true value (cm)	MDD (cm)	MDD vs mean (%)
Intra-rater reliability – agreement between 1. and 2. measurements – visit 1					
A	0.995 (0.988-0.998)	1.833	± 0.063	0.089	4.9
B	0.996 (0.992-0.997)	1.876	± 0.055	0.078	4.2
Intra-rater reliability – agreement between 1. and 2. measurements – visit 2					
A	0.997 (0.995-0.998)	1.834	± 0.053	0.075	4.1
B	0.995 (0.991-0.997)	1.884	± 0.061	0.086	4.6
Inter-rater reliability – agreement between 1. measurements – visit 1					
AB	0.939 (0.895-0.964)	1.863	± 0.210	0.297	15.9
Inter-rater reliability – agreement between means of 3 measurements – visit 1					
AB	0.944 (0.905-0.967)	1.856	± 0.201	0.285	15.4
Inter-rater reliability – agreement between 1. measurements – visit 2					
AB	0.942 (0.899-0.966)	1.859	± 0.214	0.303	16.3
Inter-rater reliability – agreement between means of 3 measurements – visit 2					
AB	0.948 (0.908-0.970)	1.856	± 0.203	0.287	15.5
Inter-day reliability – agreement between 1. measurements					
A	0.944 (0.905-0.967)	1.841	± 0.218	0.309	16.8
B	0.938 (0.895-0.963)	1.882	± 0.200	0.283	
Inter-day reliability – agreement between means of 3 measurements					15.0
A	0.950 (0.915-0.970)	1.836	± 0.208	0.294	16.0
B	0.942 (0.902-0.966)	1.875	± 0.195	0.276	14.7

CI = Confidence Interval; ICC = Intraclass Correlation Coefficient; MDD = Minimal Detectable Difference.

Supplemental Table 4 Agreement between single measurements and between means of measurements in male participants (n = 48)

Rater	ICC (95% CI)	Mean (cm)	95% CI of true value (cm)	MDD (cm)	MDD vs mean (%)
Intra-rater reliability – agreement between 1. and 2. measurements – visit 1					
A	0.999 (0.997-0.999)	2.397	± 0.042	0.060	2.5
B	0.997 (0.995-0.998)	2.432	± 0.057	0.080	3.3
Intra-rater reliability – agreement between 1. and 2. measurements – visit 2					
A	0.998 (0.995-0.999)	2.423	± 0.051	0.072	3.0
B	0.997 (0.995-0.998)	2.463	± 0.055	0.078	3.2
Inter-rater reliability – agreement between 1. measurements – visit 1					
AB	0.977 (0.958-0.987)	2.419	± 0.164	0.233	9.6
Inter-rater reliability – agreement between means of 3 measurements – visit 1					
AB	0.979 (0.961-0.989)	2.411	± 0.153	0.216	9.0
Inter-rater reliability – agreement between 1. measurements – visit 2					
AB	0.976 (0.956-0.986)	2.446	± 0.157	0.223	9.1
Inter-rater reliability – agreement between means of 3 measurements – visit 2					
AB	0.971 (0.945-0.985)	2.440	± 0.170	0.241	9.9
Inter-day reliability – agreement between 1. measurements					
A	0.967 (0.941-0.981)	2.417	± 0.190	0.269	11.1
B	0.960 (0.930-0.978)	2.448	± 0.204	0.288	11.8
Inter-day reliability – agreement between means of 3 measurements					
A	0.970 (0.946-0.983)	2.406	± 0.180	0.255	10.6
B	0.963 (0.933-0.979)	2.445	± 0.198	0.280	11.5

CI = Confidence Interval; ICC = Intraclass Correlation Coefficient; MDD = Minimal Detectable Difference.
